# Coronavirus and Exceptional Health Situations: The First Disaster With Benefits on Air Pollution

**DOI:** 10.1017/dmp.2020.174

**Published:** 2020-05-29

**Authors:** Jean-Baptiste Bouillon-Minois, François-Xavier Lesage, Jeannot Schmidt, Frédéric Dutheil

**Affiliations:** Université Clermont Auvergne, CNRS, LaPSCo, Physiological and Psychosocial Stress, University Hospital of Clermont–Ferrand, CHU Clermont–Ferrand, Emergency Medicine, F–63000 Clermont–Ferrand, France; University of Montpellier, Laboratory Epsylon EA, Dynamic of Human Abilities & Health Behaviors, CHU Montpellier, University Hospital of Montpellier, Occupational and Preventive Medicine, Montpellier, France; Université Clermont Auvergne, CNRS, LaPSCo, Physiological and Psychosocial Stress, University Hospital of Clermont–Ferrand, CHU Clermont–Ferrand, Emergency Medicine, F–63000 Clermont–Ferrand, France; Université Clermont Auvergne, CNRS, LaPSCo, Physiological and Psychosocial Stress, University Hospital of Clermont–Ferrand, CHU Clermont–Ferrand, Occupational and Environmental Medicine, WittyFit, F–63000 Clermont–Ferrand, France

**Keywords:** air pollution, COVID-19, disasters, pandemics

Since 1976 and the Seveso disaster, an industrial accident in a small chemical manufacturing facility that induced a chloracne epidemic in 447 Italian people, killed more than 80,000 animals, and exposed more than 37,000 people to 2,3,7,8-tetarchlorodibenzo-p-dioxin,^1^ the world discovered and adapted its health system to extraordinary catastrophes and their environmental consequences. Many countries can be impacted by the pollution created by a disaster; in 1986 after the Chernobyl disaster, the worst nuclear accident that occurred at reactor number 4 at the Chernobyl Nuclear Power Plant, radioactivity was discovered in Scottish people.^2,3^ Most recently, we can mention the French explosion disaster of the AZF factory in 2001 (AZF for “AZote Fertilisant”, ie, nitrogen fertilizer) with its large environmental consequences in the Garonne River (nitric acid, NH_4_, NO_3_, COT) that killed aquatic fauna. After the Fukushima disaster, the most severe nuclear accident since the 1986 Chernobyl disaster, at the Fukushima Daiichi Nuclear Power Plant in 2011, 154,000 residents were evacuated from a 20-kilometer radius zone around the nuclear reactor because of the radiation pollution.^4^

But there are disasters not caused by human activities. New emergent diseases, such as the H1N1/09 flu (a swine origin influenza A virus subtype H1N1 strain responsible for the 2009 swine flu pandemic) was declared in June 2009 by the World Health Organization as a pandemic, causing between 151,700 and 575,400 deaths around the world.^5^ The H1N1 pandemic was not accompanied by a decrease in air-pollution.

**FIGURE 1:**
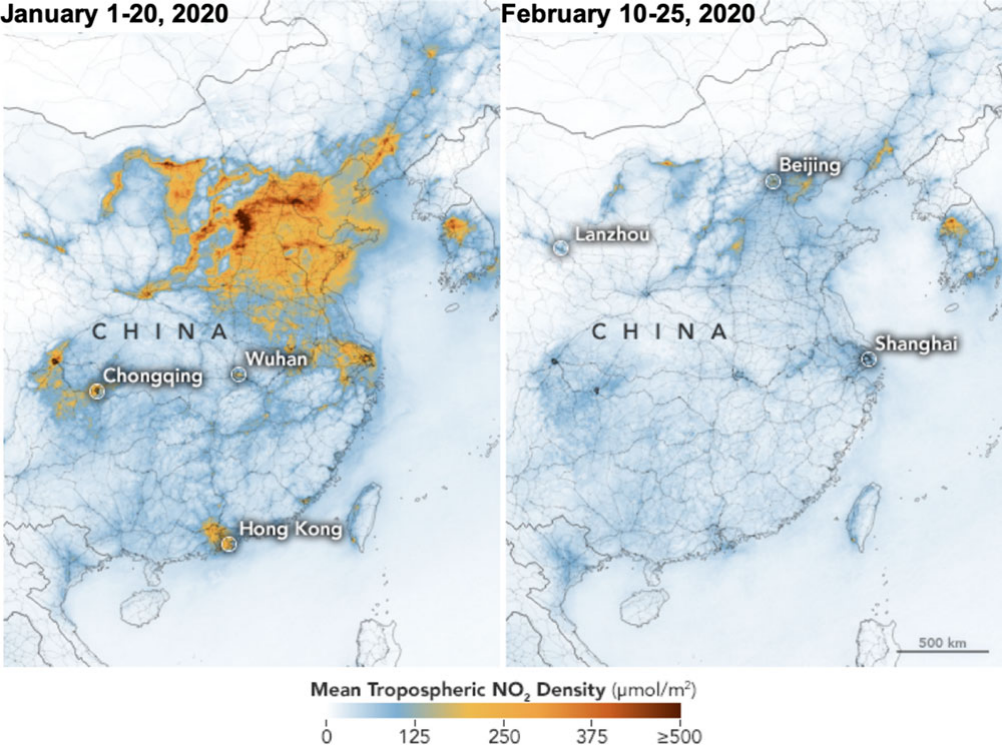
Decrease of Air Pollution in China Between January 1 and February 25 (From NASA Earth Observatory; https://earthobservatory.nasa.gov/images/146362/airborne-nitrogen-dioxide-plummets-over-china).

Since late 2019, a new highly contagious coronavirus, named severe acute respiratory syndrome coronavirus-2 (SARS-CoV-2), was describe in Wuhan, Hubei, China.^6^ The regional authorities declared quarantine status of areas infected.^7^ Consequently, human activities decreased drastically, such as industry emissions, mass transportation, and vehicle circulation. National Aeronautics and Space Administration (NASA) satellites (Tropospheric Monitoring Instrument on European Spatial Agency and Ozone Monitoring Instrument on NASA satellite) documented a massive decrease (up to 25%) in nitrogen dioxide (NO_2_) over China between January 1 and February 25 (Figure 1), and a decrease of 6% of global pollution.^8^

The regional epidemic fast became a pandemic, and authorities in countries around the world enacted massive containment measures, with more than 3.5 billion people under coronavirus restrictions (more than half the global population).^9-11^ In most major cities, air pollution decreased up to 50%, such as in Paris (-54% of NO2 air pollution).^12^ In addition to the global decrease in air pollution due to a slow-down of industry and traffic, other benefits are decreased extraction of the world’s vital resources, such as oil, increased global social interaction, and changes in behaviors toward the use of less-polluting resources, such as online activities^13^ and short supply chain.^14,15^

Before this disaster, the international community’s worries about air pollution and global warming increased daily.^16^ The SARS-CoV-2 pandemic is probably the very first disaster in the history of humanity that has resulted in a decrease in air-pollution. In addition to this benefit of the SARS-CoV-2 pandemic on decreased air pollution, public health preparedness may be enhanced both from effective disaster management protocols and from improved strategies to help communities prepare for the impact of climate change on people’s health.
